# The frequency and characteristics of ultrasonographic ankle joint involvement in systemic lupus erythematosus: A prospective cross-sectional single-center study

**DOI:** 10.1097/MD.0000000000029196

**Published:** 2022-08-05

**Authors:** Ljiljana Smiljanic Tomicevic, Alojzija Hocevar, Goran Sukara, Darija Cubelic, Miroslav Mayer

**Affiliations:** a Division of Clinical Immunology and Rheumatology, Department of Internal Medicine, University of Zagreb, School of Medicine, University Hospital Centre Zagreb, Zagreb, Croatia; b Department of Rheumatology, University Medical Centre Ljubljana, Ljubljana, Slovenia; c Medical Faculty, University of Ljubljana, Ljubljana, Slovenia.

**Keywords:** ankle, arthritis, systemic lupus erythematosus, tendons, ultrasound

## Abstract

The involvement of ankles in systemic lupus erythematosus (SLE) has not been widely studied. The aim of our prospective study was to determine the characteristics of the ankle joint and tendon involvement in SLE using ultrasound (US) as an imaging modality. Sixty consecutive patients with SLE underwent a detailed clinical evaluation and US examination. Gray-scale and power Doppler US of the bilateral tibiotalar (TT) joints, subtalar (ST) joints, and ankle tendons were performed using a multiplanar scanning technique. Joint effusion, synovitis, tenosynovitis, enthesitis, and vascularization were assessed according to the OMERACT recommendations. The Total Ankle Ultrasound Score (TAUSS) was calculated as the sum of the grades of joint effusion and synovial hypertrophy for both TT and ST joints bilaterally (ranging from 0–24) and power Doppler activity was assessed separately. Finally, US findings were correlated with physical evaluation, laboratory parameters, and SLE activity scores. US ankle joint involvement was present in 32/60 (53.3%) patients. TT joints were affected in 26 (43.3%) and ST joints in 16 (26.7%) patients. Thirteen (21.7%) patients had US tendons and/or enthesal involvement. TT joint effusion was the most frequent finding, present in 55/240 (22.9%) examined joints, followed by synovial hypertrophy detected in 18/240 (7.5%) joints. The median (interquartile range; range) TAUSS of the US-affected joints was 1 (0–2; range 1–10). There were no significant correlations between US findings and inflammatory parameters or serological parameters of disease activity, but we found a weak positive correlation between TAUSS and the European Consensus Lupus Activity Measurement (r = 0.281, *P* = .029). This study revealed a high prevalence of pathological US ankle changes in patients with SLE and a positive correlation between ankle US involvement and disease activity score (European Consensus Lupus Activity Measurement).

## 1. Introduction

Musculoskeletal involvement is one of the most common manifestations of systemic lupus erythematosus (SLE), affecting 65% to 95% of patients in the course of the disease.^[1,2]^ Although not life-threatening, joint involvement significantly affects SLE patients’ quality of life, and adequate management is important in overall patient care.^[3,4]^ As weight-bearing joints, ankles are commonly affected in various rheumatic diseases, and their pathology can lead to significant disability. Additionally, ankle pain can be caused not only by inflammatory and degenerative joint damage but also by pathological changes in tendons, bursae, entheses, or nerves. Due to the anatomical complexity of the ankle joint, especially of the subtalar (ST) joint, clinical examination is commonly inaccurate. Musculoskeletal ultrasound (US) with power Doppler (PD) has been proven as a useful imaging technique for the assessment of musculoskeletal involvement in multiple rheumatic diseases, with the ability to detect inflammation and/or structural damage of articular or periarticular structures.^[5–7]^ Interestingly, recent US studies in SLE suggested that clinical examination and laboratory testing were insufficient for early diagnosis and follow-up of musculoskeletal involvement in SLE, revealing a surprisingly high prevalence of subclinical synovitis and tendonitis.^[8,9]^

In addition, a recent study reported frequent biomechanical foot abnormalities in SLE that were not captured by a standardized assessment of disease activity.^[10]^ As previous US studies in SLE focused mainly on hand or foot small joints,^[8,9,11]^ we aimed to assess the frequency of ankle joint and tendon involvement in SLE in our cross-sectional prospective US study. Second, we aimed to determine which clinical and/or laboratory parameters, along with disease activity indices, correlate with ankle joint involvement in SLE.

## 2. Methods

### 2.1. Settings

This prospective cross-sectional study was performed at the Department of Rheumatology, University Hospital Center Zagreb, a central national hospital, a tertiary level teaching hospital, and a center of excellence for SLE in the Republic of Croatia since 2016.

### 2.2. Patients

We included 60 consecutive adult SLE patients diagnosed and followed up at our center from January 2018 to May 2019. All patients fulfilled the American College of Rheumatology classification criteria for SLE.^[12]^ Patients with SLE overlapping with rheumatoid arthritis (i.e., Rhupus syndrome) or concurrent diagnosis of other diseases affecting the joints (e.g., spondyloarthritis, gout, etc) were excluded from the study.

### 2.3. SLE assessment

US assessment was performed at a single time point during regular patient follow-up. On the day of US examination, all patients underwent extensive clinical and laboratory examinations, including evaluation of painful and swollen joints and joint deformities (44 joints, including ankles).

For laboratory assessment of C-reactive protein (CRP [mg/dL]), erythrocyte sedimentation rate (ESR [mm/h]), complement C3 and C4 (mg/L), antinuclear antibodies, and extracted nuclear antibodies including anti-dsDNA, anti-Sm, anti-SSA, anti-SSB, anti-histone, and anti-U1RNP antibodies were measured.

SLE activity was assessed using the Systemic Lupus Erythematosus Disease Activity Index 2000 (SLEDAI-2K) and the European Consensus Lupus Activity Measurement (ECLAM).^[13,14]^

### 2.4. US assessment

A single rheumatologist with more than 6 years of experience in musculoskeletal US who was blinded to the clinical and laboratory data performed the US examination. A high-resolution US with a multifrequency linear array transducer (4–15 MHz) with PD was used. To assess vascularization PD signal settings were: pulse repetition frequency 800 Hz and Doppler frequency 7.1 MHz. Color gain was set just below the level of noise. In every SLE patient, we examined the tibiotalar (TT) joint and ST joint bilaterally. In addition, 10 ankle tendons were evaluated bilaterally: tibialis anterior, extensor hallucis longus, extensor digitorum longus, tibialis posterior, flexor hallucis longus, flexor digitorum longus, peroneus longus, peroneus brevis, Achilles tendon (AT), and plantar fascia (PF).

US examination was performed in accordance with the International Guidelines for Musculoskeletal US in Rheumatology, and the presence of the following 5 elementary lesions (i.e., joint effusion, synovial hypertrophy, bone erosions, tenosynovitis, and enthesitis was evaluated according to OMERACT definitions).^[15]^

US-detected lesions were evaluated using a dichotomous score (absent/present). In addition, a semi-quantitative scale (0–3) was used for scoring joint effusion, synovial hypertrophy, and inflammatory activity (assessed with PD) as proposed by OMERACT.^[15]^ Total Ankle Ultrasound Score (TAUSS) was calculated by a summation of grades of joint effusion and synovial hypertrophy for both TT and ST joints bilaterally. The TAUSS scores ranged from 0 to 24. Hyperemia was assessed separately.

### 2.5. Statistical analysis

Continuous variables were recorded as the arithmetic mean and standard deviation (SD), and categorical variables were recorded as frequencies and relative frequencies. To compare categorical variables between groups, we used the chi-squared test. Fisher exact test was used when the expected frequency was less than 5. To compare laboratory parameters and disease activity scores between the groups, we used a non-parametric Wilcoxon 2-sample test and the Kruskal-Wallis test. A significance level of 5% was considered statistically significant. Pearson correlation analysis was used to correlate TAUSS with clinical (age, disease duration, body mass index, steroid use) and laboratory data, SLEDAI-2K, and ECLAM. Regression analyses were performed using stepwise procedures. Analyses were performed using SAS statistical package.

### 2.6. Ethics committee approval

The study was conducted in accordance with good clinical practice guidelines and the Helsinki Declaration and was approved by the hospital ethics committee (document number 8.1-16/110-2/No 02/21 AG). All patients signed an informed consent form.

## 3. Results

### 3.1. Patients’ characteristics and treatment

Of the 60 consecutive SLE patients included in our study, 93% were women, median (interquartile range [IQR]) age 41 (Q1 = 29.5, Q3 = 53.5) years, and median disease duration of 11 (Q1 4.5, Q3 18.4) years. Demographic, clinical, and laboratory data and therapy at the time of US examination are summarized in Table 1. The median SLEDAI-2K score was 4 (Q1 = 2, Q3 = 4.5), and the ECLAM score was 1 (Q1 = 1, Q3 = 2). At the time of the examination, 18 (30.0%) patients reported either musculoskeletal symptoms or abnormal clinical examination findings, whereas 42 (70.0%) patients had no musculoskeletal symptoms. Fifty-one patients (85.0%) had normal clinical ankle evaluations (no ankle pain, tenderness on palpation, or visible swelling). Of the remaining 9 patients, 4 (6.6%) reported ankle joint pain, and 5 (8.3%) patients had swelling of the ankle during the examination. One patient reported AT pain, and 1 patient had painful PF.

**Table 1:** Demographic, clinical, and serological data of all included patients, and the therapy at the time of examination.

Feature	Result at the time of examination
Female, n (%)	56 (93,3)
Female:male ratio	14:1
Age (yrs), median (IQR)	41 (25,5)
Disease duration, years, median (IQR)	11 (14,1)
Clinical examination	
Joint involvement on examination, n (%)	18 (30)
Number of painful joints on examination, mean value (range)	7 (0–30)
Number of swollen joints on examination, mean (range)	2 (0–6)
Clinically affected ankle on examination, n (%)	9 (15)
Ankle pain on examination, n (%)	3 (5)
Ankle swelling on examination, n (%)	4 (6,7)
Ankle pain and swelling on examination, n (%)	1 (0,6)
SLEDAI-2K, median (IQR)	4 (2,75)
ECLAM, median (IQR)	1 (1)
Laboratory data during examination	
CRP, mg/dL, mean value (range)	5,1 (0–57)
ESR mm/h, mean value (range)	24,2 (1–88)
ANA positivity, n (%)	56 (93,3)
• Anti-dsDNA, n (%)	33 (55)
• Anti-dsDNA, IU/dL, mean value (range)	142,2 (0–765)
Low C3, n (%)	20 (33,3)
Low C4, n (%)	16 (26,7)
Therapy	
Corticosteroids, n (%)	52 (85)
NSAIDs, n (%)	20 (33,3)
Corticosteroids (prednisone), mean daily dose, mg	12,7
Anti-malarials, n (%)	36 (60)
Anti-malarials without other DMARDs, n (%)	24 (40)
• Hydroxychloroquine, n (%)	10 (16,7)
• Chloroquine, n (%)	14 (23,3)
Disease modifying drugs	
Methotrexate, n (%)	4 (6,7)
Azathioprine, n (%)	7 (11,7)
Mycophenolate mofetil, n (%)	8 (13,3)
Cyclosporine A, n (%)	2 (3,3)
Cyclophosphamide, n (%)	3 (5)

At the time of US examination, 51 patients (85%) were treated with glucocorticoids (GC), 9 (15%) as monotherapy, and 48 (80%) in combination with various disease-modifying anti-rheumatic drugs. Thirty-six patients (60.0%) were treated with anti-malarial drugs. The mean (SD) daily prednisone equivalent dose was 12.7 (17.6) mg (range from 2.5–100 mg). The mean (SD) GC dose was 11.9 (SD 11.9) mg in a group of patients with musculoskeletal symptoms and 13.0 (SD 19.1) mg in a group without current joint involvement. The difference between the groups was not statistically significant (*P* = .599). Twenty patients (33.3%) were receiving non-steroidal anti-inflammatory drugs, 12 (28.5%) with and 8 (44.4%) without musculoskeletal symptoms, respectively, and the difference between the groups was not significant (*P* = .249).

### 3.2. Ultrasonographic findings

Using US, we detected ankle joint involvement in 32 patients (53.3%). TT joints were affected in 26 (43.3%) and ST joints in 16 (26.7%) patients. Eleven (18.3%) patients had concurrent TT and ST joint involvement. Eleven of 26 patients (42.3%) had US changes in both TT joints and 2 (12.5%) in both ST joints.

To analyze the association between the clinical examination and sonographic findings, patients were divided into 2 subgroups. Group 1 included 9 (15%) patients with current clinical symptomatic ankle involvement (pain and/or swelling), and Group 2 included 51 (85%) patients without clinical ankle involvement. US changes in ankles were observed more frequently in Group 1 (38.8%) than in Group 2 (20.5%) (*P* = .035). TT joints were significantly more frequently US affected in Group 1 than in Group 2 (*P* = .032), while there was no difference between the compared groups in US findings of ST joint involvement. The most frequent pathologic finding in the ankle joints was joint effusion, found in 32 patients (53.3%), followed by synovial hypertrophy, detected in 14 patients (23.3%). TT joint effusion was present in 26 (43.3%) patients, and ST joint effusion in 16 patients (26.7%). TT joint effusion was more frequently detected in Group 1 (77.8% of patients) compared to Group 2 (37.2%). Synovial hypertrophy of the TT and ST joints was observed in 14 (23.3%) and 1 (1.7%) patients, respectively. No bone erosion was observed in the ankle joints. A positive PD signal (grade ≥1) was detected in only 1 patient (symptomatic patient) in a single TT joint.

**Figure 1.**
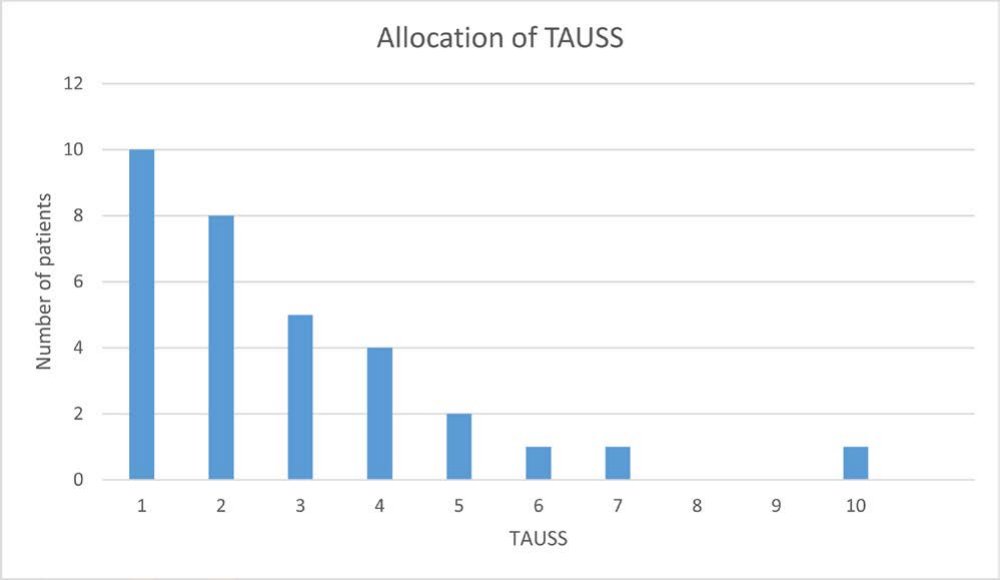
Allocation of the TAUSS in patients with SLE. SLE = systemic lupus erythematosus, TAUSS = Total Ankle Ultrasound Score.

At the articular level (240 joints in total), joint effusion was found in 55 (22.9%) and synovial hypertrophy in 18 out of 240 examined joints (7.5%). Synovial hypertrophy of grades 1, 2, and 3 was observed in 13, 5, and 0 joints, respectively. The mean (SD) calculated TAUSS was 1.5 (2.06) with a range from 0 to 10 (in patients with US ankle pathology), the median (IQR) TAUSS was 2 (1; 4). The distribution of TAUSS is shown in Figure 1.

Furthermore, 13 (21.7%) patients had US pathology of tendons and/or entheses. The PF and tibialis anterior tendon were the most commonly affected structures (in 5 [8.3%] and 4 [6.7%] patients, respectively). Of all patients, 38.3% had calcifications in the AT. Bursitis in the AT area was present in 5% of all patients, and in 1 patient, calcanear erosions were detected.

### 3.3. Ankle US findings and correlation with SLE characteristics and disease activity

We found no significant differences in demographic characteristics, disease duration time, inflammatory parameters (ESR and CRP), immuno-serological activity (dsDNA, C3, C4), or disease activity scores (SLEDAI, ECLAM) between the groups of patients with and without pathologic US findings of ankle joints and/or tendon involvement (Table 2). Significantly more patients with US-verified ankle joint involvement also had clinically swollen joints on examination.

**Table 2:** Comparison in demographic characteristics, disease duration time, inflammatory parameters, disease activity scores between the groups of patients with and without pathologic ultrasound finding of ankle joint/tendon involvement.

		Ankle joint involvement			Ankle tendon involvement		
Characteristics	All SLE n = 60	Yes 0 = 0 32	Non0 = 0 28	*P* value	Yesn = 13	No	*P* value
Age	41 (IQR 25.5)	39 (IQR 21.5)	41.5 (IQR 24.0)	.447	52 (IQR 27.0)	39 (IQR 22.0)	.073
Gender (female, %)	56 (93.3%)	31 (96.9%)	25 (89.3%)	.266	13 (100%)	43 (91.5%)	.568
Disease duration (yrs)	11 (IQR 14.1)	9.8 (IQR 9.2)	13.5 (IQR 20.7)	.238	11 (IQR 20.2)	12.6 (IQR 13.3)	.920
SLEDAI-2K	4 (IQR 2.5)	4 (IQR 2.0)	4 (IQR 3.0)	.963	5.4 (IQR 7.5)	3.6 (IQR 2.0)	.543
ECLAM	1 (IQR 1.0)	1 (IQR 1.7)	1 (IQR 1.0)	.915	1 (IQR 3.7)	1 (IQR 1.0)	.543
Tender joints (42 count)	17 (28.3%)	11 (34.4%)	6 (21.4%)	.39	3 (23.0%)	14 (29.8%)	.740
Swollen joints (42 count)	9 (15%)	8 (32%)	1 (3.6%)	.029	2 (15.4%)	7 (14.9%)	1
Ankle joint pain	4 (6.7%)	2 (6.25%)	2 (7.1%)	1	1 (7.7%)	3 (6.4%)	1
Ankle joint swollen	5 (8.3%)	5 (15.6%)	0	.055	1 (7.7%)	4 (8.5%)	1
ESR	24.2 (IQR 30.0)	23.5 (IQR 28.0)	24.9 (IQR 29.5)	.676	12.5 (IQR 21.0)	18.0 (IQR 31.0)	.727
CRP	5.04 (IQR 4.4)	3.5 (IQR 3.8)	6.8 (IQR 7.0)	.391	2.2 (IQR 2.0)	2.0 (IQR 5.8)	.814
Anti-dsDNA	33 (55%)	16 (50%)	17 (60.7%)	.405	6 (46.1%)	27 (57.4%)	.469
Low C3 or C4	23 (38.3%)	10 (31.25%)	11 (42.9%)	.515	6 (46.1%)	17 (36.2%)	.535
Glucocorticoids	51 (85%)	28 (87.5%)	23 (82.1%)	.721	11 (84.6%)	40 (85.1%)	1
Anti-malarials	32 (60.0%)	18 (56.2%)	18 (64.3%)	.526	8 (61.5%)	28 (59.6%)	.898
Other DMARDs	24 (40.0%)	16 (50.0%)	8 (33.3%)	.091	7 (53.8%)	17 (36.2%)	.249
NSAIDs	20 (33.3%)	9 (28.1%)	11 (39.3%)	.418	4 (30.8%)	16 (34.0%)	1

Nevertheless, the disease activity scores (SLEDAI and ECLAM), as well as patient age, were significantly higher in the subgroup with concurrent US pathologic findings of the ankle joint and tendon than in the subgroup with only ankle joint involvement (*P* values of *P* = .032, *P* = .033, *P* = .047, respectively).

We found no correlation between TAUSS and the patient’s age, weight, body mass index, disease duration, or any laboratory parameters investigated, or SLEDAI (Table 3). However, a positive correlation between the TAUSS and ECLAM score (r = 0.281, *P* = .029) was found. In addition, the TAUSS correlated with the prescribed GC dose (r = 0.266, *P* = .045).

**Table 3:** Pearson correlation coefficients for correlation between the TAUSS and other variables.

Variable	*P* value	R
Age (yrs)	.382	−0.115
Weight	.466	−0.095
BMI	.246	−0.152
Disease duration	.318	−0.131
Glucocorticoids	**.045**	0.266
SLEDAI-2K	.086	0.223
ECLAM	**.029**	0.281
ESR	.663	0.058
CRP	.256	−0.150
dsDNA	.305	0.134
C3	.688	−0.053
C4	.308	−0.134

## 4. Discussion

Ankle joints have so far been relatively neglected in the field of rheumatic diseases, including SLE. The anatomical complexity of the joint and deeply located synovium make clinical examination challenging and may underestimate the type and distribution of pathological changes. In particular, ST joint assessments require an experienced physician. In our study, we determined for the first time in the frequency, patterns, and characteristics of involvement of the ankle joints and tendons in SLE using US as an imaging modality. We found ankle joint involvement in more than 50% of SLE patients, and one-third of them had pathology simultaneously in the TT and ST joints. Clinically, ankle joint involvement was detected in only 15% of patients. While US changes of TT joints were significantly more frequent in patients with clinically affected ankles, US ST joint involvement was frequently clinically asymptomatic. These results are similar to those of US studies on metatarsophalangeal joints (MTPs) in SLE, which also revealed an imperfect correlation with the clinical examination.^[6,10]^ Most studies conducted to date have found a significant subclinical joint involvement of metacarpophalangeal, proximal interphalangeal, and wrist joints in patients with SLE leading to the conclusion that reliance only on physical examination of the joints may underestimate the presence of active joint inflammation.^[8,16–19]^ On the other hand, subclinical synovitis did not show a clear correlation with disease activity parameters. Although earlier studies mainly found mild changes in asymptomatic patients,^[17]^ a recent US study reported subclinical synovitis grade ≥2 in 20.8% of patients.^[16]^ The question remains whether subclinical arthritis or mild intermittent symptoms have a clinical relevance or a prognostic value.

To date, the prevalence of US ankle joint involvement in SLE has been poorly investigated. One recent study found that TT joint synovitis was significantly more common in SLE patients (25%) than in healthy controls (1.7%).^[10]^ In addition to this study, only 1 further study evaluated US ankle involvement in SLE, and their results differed significantly.^[10,18]^ Morales-Lozano et al^[10]^ reported a 25% of US ankle joint involvement, while only 0.8% of patients had US ankle pathology in a study by Salliot et al.^[18]^

Although frequent, ankle joint involvement was commonly mild in our SLE cohort, also reflected in a relatively low TAUSS (representing a sum of grades of joint effusion, synovial hypertrophy, for both TT and ST joints bilaterally). The mean TAUSS score was 1.5. The most common pathological finding in TT joints was JE, followed by SH, mostly grade 1. Since the previously reported studies did not evaluate pathological changes according to grades of severity, our results cannot be compared.^[10,18]^ The degree of synovitis is a strength of our study. A positive PD signal in the ankles was exceptionally rare in our SLE cohort. The reason could be a relatively deep-lying ankle joint and a high median maintenance GC dose. Our findings are consistent with those of other studies.^[10,18]^

Contrary to previous reports where the frequency of bone erosion was variable, we did not find erosive arthritis in TT or ST joints.^[9,18,20]^ A possible explanation could be that Rhupus patients and patients with SLE overlapping with other articular diseases were excluded from the study. We believe that the inclusion of a highly selected SLE population represents an additional strength of the study, enabling us to obtain a more credible insight into the frequency of erosive ankle joint disease in SLE.

US evidence of the involvement of tendons in the ankle area was rare in our patients with SLE. The PF is the most commonly affected tendon. Our results are in line with those of a study by Morales-Lozano et al.^[10]^

The musculoskeletal system is poorly represented in SLE activity indices. In addition, data on the correlation between US findings and SLE activity parameters are conflicting. Only a few studies found a positive correlation between hand and wrist synovitis and the SLEDAI score,^[9]^ while most studies found no correlation.^[6,19]^ However, other studies found a positive correlation between the US score for synovitis and inflammatory parameters or the anti-dsDNA titer.^[6,17]^

We found no significant differences in either measured inflammatory parameters or disease activity scores between groups of patients with and without US ankle joint involvement and/or tendon involvement. Furthermore, we detected no significant impact of disease duration on the prevalence of US ankle changes in our patients with SLE. Interestingly, TAUSS correlated weakly positively with the disease activity index ECLAM (*P* = .031; R 0.278), but not with individual laboratory parameters of disease activity (CRP, ESR, dsDNA, C3, C4).

Our study has several limitations. The drawbacks are the single-center study design and the relatively short duration of the study with a limited number of patients. Having information on physical activity before US evaluation and a follow-up data of our SLE patients would further increase the value of the study.

## 5. Conclusion

Our prospective, cross-sectional US study revealed a high prevalence of pathological changes in the ankles of patients with SLE and a weak correlation between the extensiveness and severity of ankle joint involvement (TAUSS) and overall disease activity score ECLAM.

## Author contributions

Conceptualization: Alojzija Hocevar, Ljiljana Smiljanic Tomicevic, Miroslav Mayer.

Data curation: Darija Cubelic, Ljiljana Smiljanic Tomicevic.

Formal analysis: Alojzija Hocevar.

Funding acquisition: Miroslav Mayer.

Investigation: Darija Cubelic, Goran Sukara, Ljiljana Smiljanic Tomicevic.

Supervision: Alojzija Hocevar, Ljiljana Smiljanic Tomicevic, Miroslav Mayer.

Validation: Ljiljana Smiljanic Tomicevic, Miroslav Mayer.

Writing – review & editing: Alojzija Hocevar, Ljiljana Smiljanic Tomicevic.

Writing – review & editing: Ljiljana Smiljanic Tomicevic.
